# Supplementary material for: Time-dependent memory transformation in hippocampus and neocortex is semantic in nature
Source: Nat Commun. 2023 Sep 27;14:6037. doi: 10.1038/s41467-023-41648-1 (PMC10533832; doi:10.1038/s41467-023-41648-1)
Supplement: Supplementary file 1 — Supplementary Information [file 41467_2023_41648_MOESM1_ESM.pdf]

Supplementary Information

Time-dependent memory transformation in hippocampus and neocortex is semantic in nature

Valentina Krenz[1], Arjen Alink[2,3], Tobias Sommer[3], Benno Roozendaal[4,5], and Lars Schwabe[1*]

[1]Department of Cognitive Psychology, Institute of Psychology, Universität Hamburg, Von-Melle-Park 5, 20146 Hamburg, Germany

[2]Department of General Psychology, Institute of Psychology, Universität Hamburg, Von-Melle-Park 11, 20146 Hamburg, Germany

[3]University Medical Centre Hamburg-Eppendorf, Department of Systems Neuroscience, Martinistraße 52, 20246, Hamburg, Germany

[4]Department of Cognitive Neuroscience, Radboud university medical center, 6500 HB Nijmegen, The Netherlands

[5]Donders Institute for Brain, Cognition and Behaviour, Radboud University, Kapittelweg 29, 6525 EN Nijmegen, The Netherlands

* Lars Schwabe, Department of Cognitive Psychology, Institute of Psychology, Universität Hamburg, Von-Melle-Park 5, 20146 Hamburg, Germany. Phone: +49-40-428385950. *E*-Mail: lars.schwabe@uni-hamburg.de

Supplementary Figures

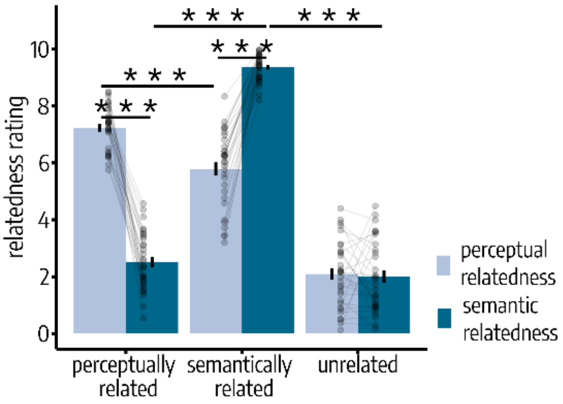

Supplementary Figure 1. **Results of the behavioral pilot.** Semantically related pictures were rated as being significantly more semantically related to the original picture (M = 9.38, SEM = 0.08) than both perceptually related (M = 2.38, SEM = 0.18; paired *t*-test *t*(31) = 33.24, *p* = 8*e*-14, *d* = 5.64, 95% Confidence Interval = [5.31, 5.97]) and unrelated pictures (M = 1.97, SEM = 0.22; paired *t*-test: *t*(31) = -29.63, *p* = 8*e*-14, *d* = -4.84, 95% Confidence Interval = [-5.16, -4.52]; main effect lure type for semantic relatedness: *F*(1.59, 49.35) = 794.01, *p* = 3*e*-36 , η_p^2 = 0.96, 95% Confidence Interval: [0.95, 0.98]). Moreover, perceptually related items were rated as being significantly more perceptually related (M = 7.22, SEM = 0.14) to original pictures than both semantically related items (M = 5.66, SEM = 0.24; paired *t*-test: *t*(31) = -6.32, *p* = 1*e*-06, *d* = -1.1, 95% Confidence Interval = [-1.44, -0.76]) and unrelated items (M = 2.05, SEM = 0.20; paired *t*-test: *t*(31) = -14.91, *p* = 3*e*-15, *d* = -2.57, 95% Confidence Interval = [-2.91, -2.23]; main effect lure type for perceptual relatedness: *F*(1.70, 52.68) = 284.85, *p* = 3*e*-36, η_p^2 = 0.90, 95% Confidence Interval: [0.86, 0.94]). Bars represent mean ± SEM. Connected dots represent individual data points. All *n* = 52 participants. All post-hoc tests were applied on estimated marginal means with Šidák correction for multiple comparisons. All reported *p*-values are two-tailed. Source data are provided as Source Data file. ****p*<0.001.

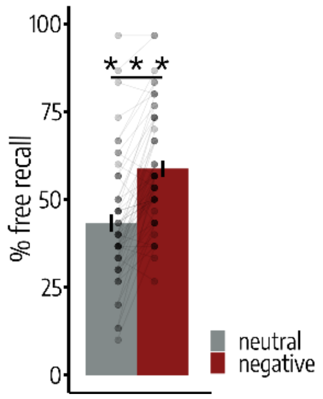

Supplementary Figure 2. **Emotional enhancement of immediate free recall.** Immediately after encoding, participants recalled significantly more often emotionally negative than neutral items (main effect emotion: $F(1, 50) = 69.33$, $p = 5e\text{-}11$, $\eta_p^2 = 0.58$, 95% Confidence Interval: [0.42, 0.72]). This analysis did not indicate a statistically significant difference between delay groups in immediate memory performance (main effect delay: $F(1, 50) = 0.17$, $p = 0.678$, $\eta_p^2 = 0.004$, 95% Confidence Interval: [2*e*-05, 0.11]; delay × emotion: $F(1, 50) = 1.13$, $p = 0.293$, $\eta_p^2 = 0.02$, 95% Confidence Interval: [6*e*-05, 0.16]; mixed ANOVA). Bars represent mean ± SEM. Connected dots represent individual data points. $N = 52$ participants. All reported *p*-values are two-tailed. Source data are provided as Source Data file. ***$p < 0.001$.

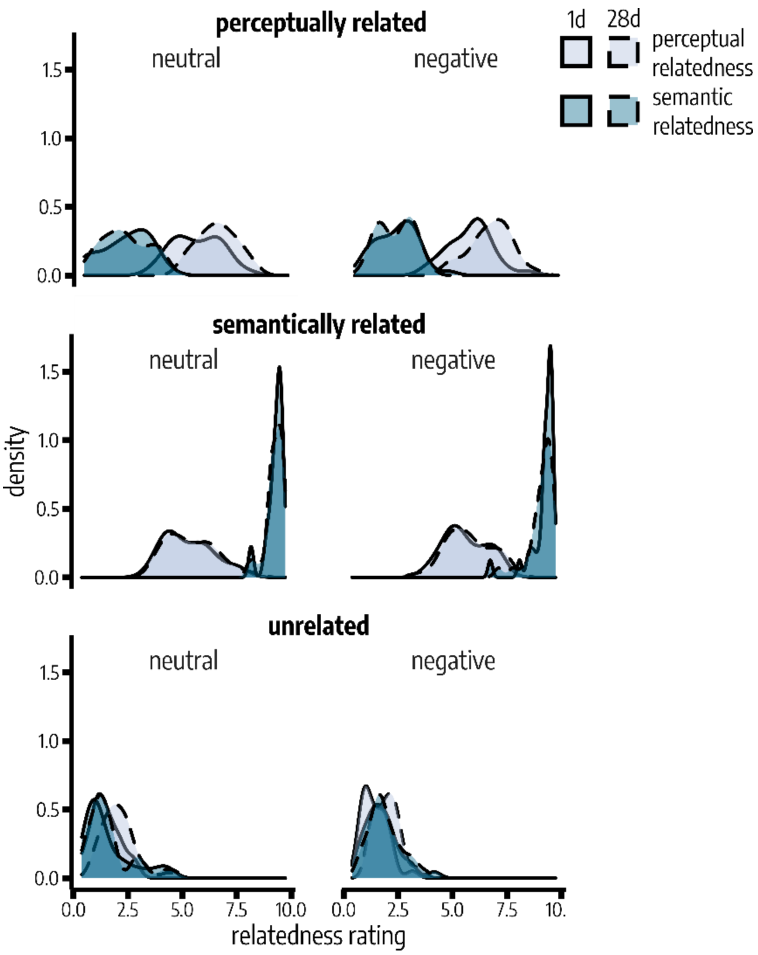

Supplementary Figure 3. **Distribution of semantic and perceptual relatedness ratings on Day 3, separately for each emotion, lure type and delay group.** Perceptually related lures were rated as significantly higher in perceptual than in semantic relatedness to their corresponding old image (paired *t*-test: *t*(51) 16.67, *p* = 3*e*-22, *d* = 3.25, 95% Confidence Interval = [2.66, 3.83]), while semantically related items were rated as significantly more semantically than perceptually related to their corresponding old image (paired *t*-test: *t*(51) = -16.38, *p* = 6*e*-22, *d* = -2.83, 95% Confidence Interval = [-3.37, -2.28]). Unrelated images were rated low in perceptual (M = 1.74, SEM = 0.17) as well as semantic relatedness (M = 1.72, SEM = 0.16) without a significant difference between rating scales *t*(51) = -0.16, p = 0.872, *d* = -0.02, 95% Confidence Interval = [-0.4, 0.37]. *N* = 52 participants. All reported *p*-values are two-tailed. Source data are provided as Source Data file.

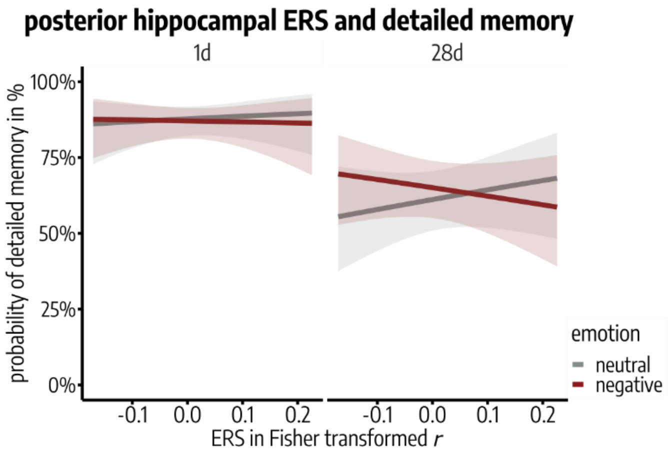

Supplementary Figure 4. **Posterior hippocampal Encoding-Retrieval-Similarity (ERS) was not associated with detailed memory representations.** Analyzing the probability of a detailed recognition (correct response for old items without false alarms for related items) by means of a binomial generalized linear mixed model with the factors left posterior hippocampal ERS, delay and emotion did not show a significant association of ERS with detailed memory (all $p > 0.688$; $n = 52$). Lines represent the predicted probability for a detailed memory with 95% Confidence Interval. All reported *p*-values are two-tailed. Source data are provided as Source Data file.

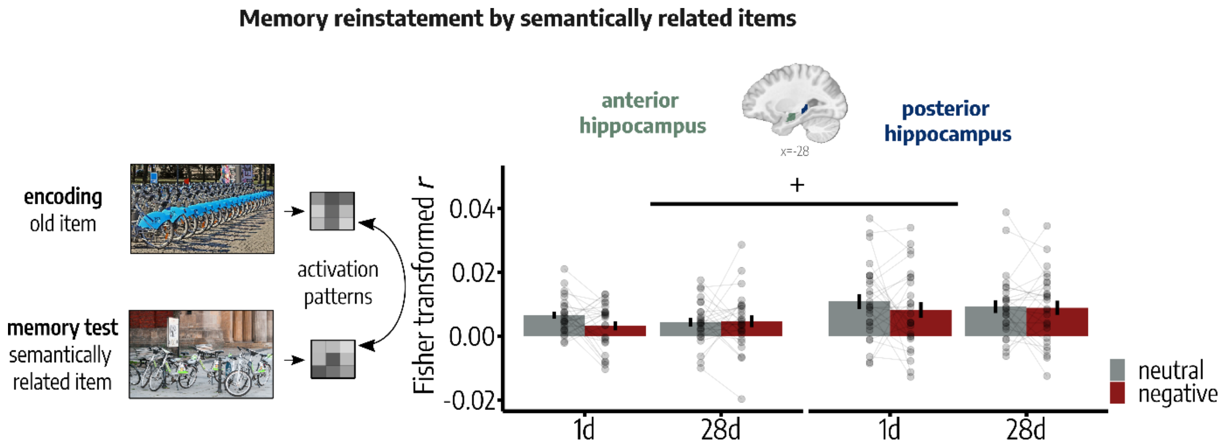

Supplementary Figure 5. **Hippocampal pattern similarities between original items at encoding and corresponding semantically related lures at memory test.** The posterior hippocampus tended to show a higher reinstatement of the semantic gist of the original memory compared to the anterior hippocampus (main effect long axis: $t(6124) = 1.94$, $p = 0.052$, $\beta = 0.004$, 95% Confidence Interval = [-4e-05, 0.01]; linear mixed model, LMM). No effect approached significance when analyzing hippocampal reinstatement of the perceptual gist (all $p > 0.235$; LMM). Bars represent mean ± SEM. Connected dots represent individual data points. $N = 52$ participants. All reported p-values are two-tailed. All depicted images are licensed under Creative Commons BY-SA License: image representing 'old' item is courtesy of W. Bulach (https://commons.wikimedia.org/wiki/File:00_2141_Bicycle-sharing_systems_-_Sweden.jpg; edited), image representing 'semantically related' item is courtesy of Matti Blume (https://commons.wikimedia.org/wiki/File:Bike_share_2019,_Berlin_(P1080139).jpg; edited). Regions of interest are visualized on a sagittal section of a T1-weighted template[1] in MNI-152 space. Source data are provided as Source Data file. $^{+}p < 0.060$.

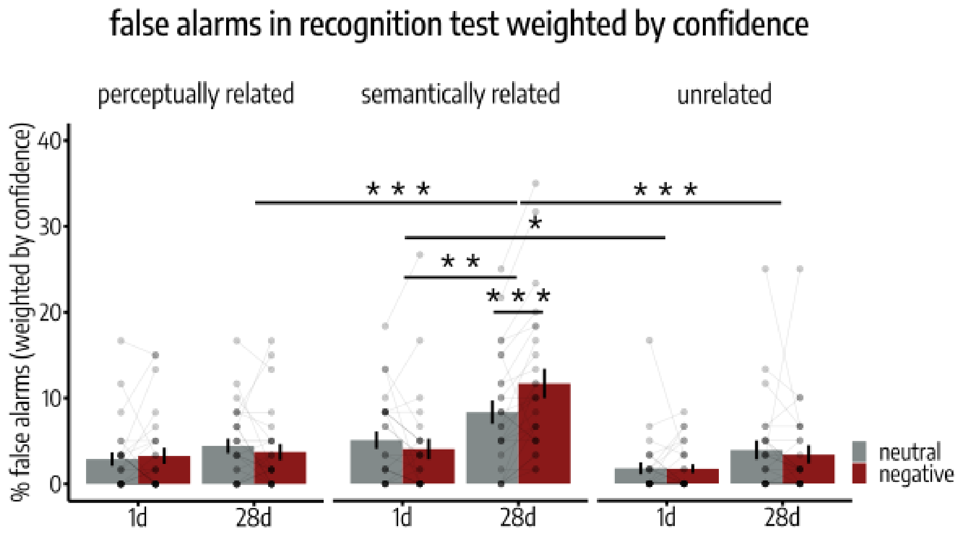

Supplementary Figure 6. **Time dependent changes in false alarms weighted by confidence.** The increase in false alarms (FAs) from 1d to 28d after encoding was significantly higher for lures that were semantically related to the encoded pictures, compared to perceptually related (interaction contrast: $t(50) = -3.89$, $p = 0.001$, $d = -0.6$, 95% Confidence Interval = [-0.9, -0.3]) or unrelated lures (interaction $t(50) = -2.61$, p = 0.035, $d = -0.48$, 95% Confidence Interval = [-0.83, -0.12]; delay × lure type: $F(1.50, 75.19) = 8.83$, $p = 0.001$, $\eta_p^2 = 0.15$, 95% Confidence Interval: [0.04, 0.32]; main effect lure type: $F(1.50, 75.19) = 37.45$, $p = 3e\text{-}10$, $\eta_p^2 = 0.43$, 95% Confidence Interval: [0.28, 0.58]). This semantization of memories over time was significantly higher for emotionally negative compared to neutral items (delay × lure type × emotion: $F(1.96, 98.12) = 5.57$, $p = 0.005$, $\eta_p^2 = 0.10$, 95% Confidence Interval: [0.02, 0.24]; mixed ANOVA). Bars represent mean ± SEM. Connected dots represent individual data points. $N = 52$ participants. All reported p-values are two-tailed. Source data are provided as Source Data file. ***$p < 0.001$; **$p < 0.010$; *$p < 0.050$.

Supplementary Tables

Supplementary Table 1. **Responses on Day 2 for each of the 30 stimuli per emotion and item category.**

	1d		28d	
**response category**	**neutral**	**negative**	**neutral**	**negative**
**old**				
hit	27.73 (0.52)	27.38 (0.43)	21.69 (1.13)	23.65 (0.93)
miss	2.12 (0.50)	2.24 (0.41)	8.19 (1.11)	6.12 (0.89)
no response	0.15 (0.07)	0.19 (0.08)	0.12 (0.12)	0.23 (0.16)
**perceptually related**				
correct rejection	28.69 (0.34)	28.54 (0.37)	27.88 (0.39)	27.85 (0.50)
false alarm	1.31 (0.34)	1.38 (0.37)	2.00 (0.36)	1.85 (0.42)
no response	-	0.08 (0.08)	0.12 (0.12)	0.31 (0.17)
**semantically related**				
correct rejection	27.69 (0.46)	28.27 (0.49)	26.00 (0.62)	24.96 (0.62)
false alarm	2.27 (0.45)	1.73 (0.49)	3.85 (0.59)	4.92 (0.62)
no response	0.04 (0.04)	-	0.15 (0.09)	0.12 (0.08)
**unrelated**				
correct rejection	29.15 (0.26)	29.15 (0.26)	27.92 (1.88)	28.35 (0.43)
false alarm	0.77 (0.24)	0.81 (0.27)	1.88 (9.50)	1.54 (0.40)
no response	0.08 (0.05)	0.04 (0.04)	0.19 (0.10)	0.12 (0.06)

Data represents mean (SEM). Source data are provided as Source Data file.

Supplementary Table 2. **Analyses of confidence in false alarms for each lure type.**

**parameters for generalized linear mixed models**				
**semantically related lures**				
**fixed effects**	*z*	*p*	*β*	95% CI
intercept	-2.21	0.027	-0.76	-1.43, -0.09
delay	-0.66	0.506	-0.28	-1.12, 0.55
emotion	0.35	0.725	0.16	-0.75, 1.07
delay × emotion	0.75	0.455	0.41	-0.66, 1.47
**random effects**	variance	SD	*n*	
participant (intercept)	0.31	0.55	46	
stimulus (intercept)	0.15	0.39	58	
marginal R^2 / conditional R^2: 0.01 / 0.13				
**perceptually related lures**				
**fixed effects**	*z*	*p*	*β*	95% CI
intercept	-1.77	0.077	-0.93	-1.95, 0.1
delay	-0.22	0.828	-0.14	-1.41, 1.13
emotion	0.68	0.497	0.42	-0.79, 1.62
delay × emotion	-1.51	0.131	-1.21	-2.79, 0.36
**random effects**	variance	SD	*n*	
participant (intercept)	0.9	0.95	39	
stimulus (intercept)	0.25	0.50	50	
marginal R^2 / conditional R^2: 0.05 / 0.3				
**unrelated lures**				
**fixed effects**	*z*	*p*	*β*	95% CI
intercept	-1.08	0.28	-0.85	-2.4, 0.69
delay	-0.76	0.449	-0.73	-2.63, 1.16
emotion	-0.16	0.873	-0.15	-1.96, 1.67
delay × emotion	0.26	0.792	0.29	-1.87, 2.46
**random effects**	variance	SD	*n*	
participant (intercept)	2.31	1.52	31	
marginal R^2 / conditional R^2: 0.01 / 0.42				

Note that all models were fitted on the whole data set (52 participants and 60 lures). Any discrepancies in *n* are due to an insufficient number of cases (false alarms) in a specific condition. Source data are provided as Source Data file.

Supplementary Table 3. **Average number of stimulus sets that were perceptually transformed, semantically transformed, detailed or entirely forgotten on Day 2, separately for each emotionality and delay group.**

	1d		28d	
**specificity category**	**neutral**	**negative**	**neutral**	**negative**
**perceptually transformed**	1.04 (0.27)	1.19 (0.31)	1.35 (0.28)	1.08 (0.27)
**semantically transformed**	2.00 (0.40)	1.54 (0.41)	3.15 (0.47)	4.15 (0.47)
**forgotten**	1.58 (0.29)	2.15 (0.40)	6.85 (0.95)	5.08 (0.81)
**detailed**	24.96 (0.87)	24.65 (0.80)	17.69 (0.95)	18.38 (1.18)

Data represents mean (SEM) number of stimulus sets per category. Source data are provided as Source Data file.

Supplementary Table 4. **Analyses of the probability for a detailed, forgotten, semantically or perceptually transformed stimulus on Day 2.**

**parameters for generalized linear mixed models**				
**detailed**				
**fixed effects**	*z*	*p*	*β*	95% CI
intercept	8.69	4*e*-18	1.97	1.53, 2.42
delay	-5.29	1*e*-07	-1.51	-2.07, -0.95
emotion	-0.35	0.723	-0.07	-0.44, 0.31
delay × emotion	1.13	0.258	0.21	-0.15, 0.57
**random effects**	variance	SD	*n*	
participant (intercept)	0.81	0.9	52	
stimulus set (intercept)	0.23	0.48	60	
marginal R^2 / conditional R^2: 0.10 / 0.32				
**forgotten**				
**fixed effects**	*z*	*p*	*β*	95% CI
intercept	-12.31	8*e*-35	-3.26	-3.78, -2.74
delay	5.75	9*e*-09	1.79	1.18, 2.41
emotion	1.22	0.223	0.31	-0.19, 0.82
delay × emotion	-3.00	0.003	-0.75	-1.25, -0.26
**random effects**	variance	SD	*n*	
participant (intercept)	0.78	0.88	52	
stimulus set (intercept)	0.31	0.56	60	
marginal R^2 / conditional R^2: 0.11 / 0.33				
**semantically transformed**				
**fixed effects**	*z*	*p*	*β*	95% CI
intercept	-11.78	5*e*-32	-3.12	-3.64, -2.6
delay	2.17	0.03	0.64	0.06, 1.22
emotion	-1.2	0.23	-0.35	-0.92, 0.22
delay × emotion	2.46	0.014	0.66	0.14, 1.19
**random effects**	variance	SD	*n*	
participant (intercept)	0.63	0.79	52	
stimulus set (intercept)	0.52	0.72	60	
marginal R^2 / conditional R^2: 0.06 / 0.3				
**perceptually transformed**				
**fixed effects**	*z*	*p*	*β*	95% CI
intercept	-11.18	5*e*-29	-4.19	-4.93, -3.46
delay	1.02	0.308	0.42	-0.38, 1.22
emotion	0.61	0.542	0.23	-0.51, 0.97
delay × emotion	-1.05	0.294	-0.41	-1.18, 0.36
**random effects**	variance	SD	*n*	
participant (intercept)	1.00	1.00	52	
stimulus set (intercept)	0.79	0.89	60	
marginal R^2 / conditional R^2: 0.004 / 0.35				

Source data are provided as Source Data file.

Supplementary Table 5. **Relatedness ratings on Day 3.**

lure type	1d perceptual relatedness	1d semantic relatedness	28d perceptual relatedness	28d semantic relatedness
**perceptually related**				
neutral	5.56 (0.32)	2.32 (0.22)	6.48 (0.25)	2.27 (0.25)
negative	5.71 (0.34)	2.34 (0.22)	6.63 (0.23)	2.28 (0.26)
**semantically related**				
neutral	5.27 (0.33)	9.27 (0.12)	5.37 (0.27)	9.25 (0.17)
negative	5.60 (0.31)	9.19 (0.11)	5.76 (0.25)	9.11 (0.17)
**unrelated**				
neutral	1.47 (0.22)	1.57 (0.20)	2.05 (0.27)	1.65 (0.22)
negative	1.43 (0.23)	1.77 (0.26)	1.99 (0.25)	1.88 (0.26)

Data represents mean (SEM). Source data are provided as Source Data file.

Supplementary Table 6. **Analysis of false alarms depending on individual rating of semantic and perceptual relatedness between encoded items and lures.**

parameters for generalized linear mixed				
**fixed effects**	*z*	*p*	*β*	95% CI
intercept	-14.13	3*e*-45	-3.95	-4.50, -3.40
delay	2.40	0.016	0.85	0.16, 1.55
semantic rel.	2.04	0.041	0.07	0.003, 0.13
perceptual rel.	0.78	0.435	0.03	-0.05, 0.11
emotion	0.05	0.964	0.009	-0.39, 0.41
delay × semantic rel.	-0.69	0.490	-0.02	-0.09, 0.04
delay × perceptual rel.	0.45	0.651	0.02	-0.07, 0.12
semantic rel. × perceptual rel.	0.61	0.539	0.006	-0.01, 0.02
delay × emotion	0.16	0.870	0.03	-0.36, 0.42
semantic rel. × emotion	-0.48	0.629	-0.02	-0.11, 0.07
perceptual rel. × emotion	-0.25	0.799	-0.01	-0.13, 0.10
delay × semantic rel. × perceptual rel.	1.52	0.128	0.02	-0.005, 0.04
delay × semantic rel. × emotion	2.36	0.018	0.12	0.02, 0.21
delay × perceptual rel. × emotion	-0.01	0.991	-7*e*-04	-0.13, 0.13
semantic rel. × perceptual rel. × emotion	-0.92	0.360	-0.01	-0.04, 0.01
delay × semantic rel. × perceptual rel. × emotion	0.07	0.940	0.001	-0.03, 0.03
**random effects**	variance	SD	*n*	
participant (intercept)	1.28	1.13	52	
stimulus (intercept)	0.64	0.80	180	
marginal R^2 / conditional R^2: 0.07 / 0.41				

Source data are provided as Source Data file. Semantic rel. = group mean centered semantic relatedness rating between a lure and its corresponding original stimulus; perceptual rel. = group mean centered perceptual relatedness rating between a lure and its corresponding original stimulus.

Supplementary Table 7. **Analysis of false alarms for semantically related lures depending on the level of perceptual relatedness to their corresponding original item.**

**parameters for generalized linear mixed model**				
**fixed effects**	z	p	β	95% CI
intercept	-9.81	1*e*-22	-3.16	-3.8, -2.53
perceptual rel. level	0.71	0.476	0.21	-0.37, 0.8
delay	1.23	0.218	0.49	-0.29, 1.26
emotion	-1.05	0.291	-0.41	-1.17, 0.35
perceptual rel. level × delay	1.24	0.215	0.47	-0.28, 1.22
perceptual rel. level × emotion	0.13	0.898	0.06	-0.81, 0.92
delay × emotion	2.19	0.029	0.93	0.1, 1.76
perceptual rel. level × delay × emotion	-0.86	0.392	-0.46	-1.51, 0.59
**random effects**	variance	SD	n	
participant (intercept)	0.87	0.93	52	
stimulus (intercept)	0.45	0.67	60	
marginal R^2 / conditional R^2: 0.07 / 0.34				

Perceptual relatedness (perceptual rel. level) level represents low (≤ 5) vs. high (> 5) rating in perceptual relatedness of a semantically related stimulus to its corresponding lure. Source data are provided as Source Data file.

Supplementary Table 8. **Analysis of delay-dependent changes in the anterior-posterior axis in Encoding-Retrieval-Similarity.**

parameters for linear mixed model				
**fixed effects**	*t* (df)	p_{corr}	β	95% CI
intercept	2.68 (279.33)	0.016	0.005	0.001, 0.01
delay	-1.31 (352.02)	0.381	-0.003	-0.01, 0.002
emotion	-1.53 (687.11)	0.253	-0.003	-0.01, 0.001
long axis	0.04 (6124)	1.931	1*e*-04	-0.004, 0.004
delay × emotion	1.78 (6124)	0.149	0.01	-0.001, 0.01
delay × long axis	2.35 (6124)	0.038	0.01	0.001, 0.01
emotion × long axis	1.47 (6124)	0.283	0.005	-0.002, 0.01
delay × emotion × long axis	-0.7 (6124)	0.974	-0.005	-0.01, 0.01
**random effects**	variance	SD	*n*	
participant (intercept)	1*e*-05	0.003	52	
stimulus set (intercept)	3*e*-06	0.002	60	
marginal R^2 / conditional R^2: 0.07 / 0.34				

Source data are provided as Source Data file. p_{corr} = Bonferroni corrected *p*-values.

Supplementary Table 9. **Analysis of delay-dependent changes in the anterior-posterior axis in Encoding-Retrieval-Similarity including only trials with correct recognition (hits).**

parameters for linear mixed model				
**fixed effects**	*t* (df)	p_{corr}	β	95% CI
intercept	2.66 (225.68)	0.017	0.005	0.001, 0.01
delay	-1.79 (332.73)	0.148	-0.005	-0.01, 4*e*-04
emotion	-1.63 (455.09)	0.209	-0.004	-0.01, 0.001
long axis	-0.41 (5107.81)	1.358	-0.001	-0.01, 0.004
delay × emotion	1.91 (5134.11)	0.112	0.01	-2*e*-04, 0.01
delay × long axis	3.13 (5107.81)	0.004	0.01	0.004, 0.02
emotion × long axis	1.62 (5107.81)	0.209	0.01	-0.001, 0.01
delay × emotion × long axis	-1.32 (5107.81)	0.372	-0.01	-0.02, 0.003
**random effects**	variance	SD	*n*	
participant (intercept)	2*e*-05	0.004	52	
stimulus set (intercept)	9*e*-06	0.003	60	
marginal R^2 / conditional R^2: 0.07 / 0.34				

Source data are provided as Source Data file. p_{corr} = Bonferroni corrected *p*-values.

Supplementary Table 10. **Analysis of association between left posterior hippocampal Encoding-Retrieval-Similarity (ERS) and correct recognition, i.e. hits.**

parameters for generalized linear mixed model				
**fixed effects**	*z*	*p*	*β*	95% CI
intercept	11.28	2*e*-29	3.03	2.51, 3.56
ERS	-1.61	0.108	-4.51	-10.02, 1.00
emotion	-0.44	0.662	-0.11	-0.61, 0.39
delay	-5.72	1*e*-08	-1.83	-2.46, -1.21
ERS × emotion	0.09	0.925	0.37	-7.34, 8.07
ERS × delay	2.18	0.030	7.31	0.73, 13.89
emotion × delay	2.71	0.007	0.65	0.18, 1.13
ERS × emotion × delay	-1.23	0.218	-5.81	-15.06, 3.43
**random effects**	variance	SD	*n*	
participant (intercept)	0.91	0.95	52	
stimulus set (intercept)	0.35	0.59	60	
marginal R^2 / conditional R^2: 0.12 / 0.36				

Source data are provided as Source Data file.

Supplementary Table 11. **Analysis of association between left posterior hippocampal Encoding-Retrieval-Similarity (ERS) and detailed memory performance.**

**parameters for generalized linear mixed model**				
**fixed effects**	*z*	*p*	*β*	95% CI
intercept	8.66	5*e*-18	1.97	1.52, 2.42
ERS	0.4	0.689	0.84	-3.26, 4.94
emotion	-0.32	0.747	-0.06	-0.44, 0.31
delay	-5.3	1*e*-07	-1.52	-2.08, -0.96
ERS × emotion	-0.38	0.705	-1.13	-7.01, 4.74
ERS × delay	0.2	0.845	0.53	-4.75, 5.8
emotion × delay	1.23	0.219	0.23	-0.14, 0.6
ERS × emotion × delay	-0.38	0.705	-1.44	-8.86, 5.99
**random effects**	variance	SD	*n*	
participant (intercept)	0.81	0.9	52	
stimulus set (intercept)	0.23	0.48	60	
marginal R^2 / conditional R^2: 0.1 / 0.32				

Source data are provided as Source Data file.

Supplementary Table 12. **Analyses of association between left posterior hippocampal Encoding-Retrieval-Similarity (ERS) and memory specificity, i.e. false alarms for semantically related and perceptually related lures.**

**parameters for generalized linear mixed model**				
**semantically related lures**				
**fixed effects**	*z*	*p*	*β*	95% CI
intercept	-10.88	1*e*-27	-3.08	-3.63, -2.52
ERS	-1.22	0.223	-3.41	-8.9, 2.08
emotion	-1.31	0.19	-0.37	-0.92, 0.18
delay	2.22	0.026	0.73	0.09, 1.38
ERS × emotion	1.01	0.312	4.44	-4.17, 13.04
ERS × delay	1.97	0.049	7.16	0.02, 14.29
emotion × delay	2.71	0.007	0.71	0.19, 1.22
ERS × emotion × delay	-1.45	0.146	-7.78	-18.26, 2.71
**random effects**	variance	SD	*n*	
participant (intercept)	0.91	0.95	52	
stimulus set (intercept)	0.5	0.71	60	
marginal R^2 / conditional R^2: 0.07 / 0.35				
**perceptually related lures**				
**fixed effects**	*z*	*p*	*β*	95% CI
intercept	-10.75	6*e*-27	-4.07	-4.81, -3.32
ERS	-1.33	0.184	-5.26	-13.02, 2.5
emotion	0.52	0.606	0.19	-0.53, 0.91
delay	1.51	0.132	0.63	-0.19, 1.44
ERS × emotion	-0.12	0.905	-0.66	-11.61, 10.29
ERS × delay	0.50	0.619	2.60	-7.64, 12.83
emotion × delay	-0.57	0.569	-0.20	-0.88, 0.48
ERS × emotion × delay	0.78	0.434	5.63	-8.46, 19.71
**random effects**	variance	SD	*n*	
participant (intercept)	1.26	1.12	52	
stimulus set (intercept)	0.84	0.92	60	
marginal R^2 / conditional R^2: 0.02 / 0.40				

Source data are provided as Source Data file.

Supplementary Table 13. **Analysis of association between left posterior hippocampal Encoding-Retrieval-Similarity (ERS) and response time during recognition testing.**

	parameters for linear mixed model			
**fixed effects**	*t* (df)	*p*	*β*	95% CI
intercept	18.07 (61.17)	3*e*-26	525.53	468.51, 582.54
ERS	0.15 (3060.25)	0.883	33.81	-417.43, 485.05
delay	0.81 (58.82)	0.422	32.88	-46.75, 112.52
emotion	0.59 (176.09)	0.556	10.13	-23.52, 43.79
ERS × delay	-0.52 (3071.11)	0.603	-171.25	-816.75, 474.25
ERS × emotion	0.46 (3065.52)	0.644	151.79	-492.65, 796.23
delay × emotion	-1.32 (3007.11)	0.187	-29.77	-74.00, 14.46
ERS × delay × emotion	0.19 (3069.05)	0.851	86.27	-815.23, 987.78
**random effects**	variance	SD	*n*	
participant (intercept)	18177.38	134.82	52	
stimulus set (intercept)	670.65	25.9	60	
marginal R^2 / conditional R^2: 0.1 / 0.32				

Source data are provided as Source Data file.

Supplementary Table 14. **Analyses of delay-dependent changes in anterior-posterior hippocampal representational similarity between original items at encoding and corresponding related lures during recognition testing.**

**parameters for linear mixed model**				
**semantically related lures**				
**fixed effects**	*t* (df)	*p*	*β*	95% CI
intercept	3.61 (263)	0.0004	0.01	0.003, 0.01
delay	-0.85 (319.68)	0.396	-0.002	-0.01, 0.003
emotion	-1.44 (660.89)	0.151	-0.003	-0.01, 0.001
long axis	1.94 (6124)	0.052	0.004	-4*e*-05, 0.01
delay × emotion	1.13 (6124)	0.26	0.004	-0.003, 0.01
delay × long axis	0.16 (6124)	0.869	0.001	-0.01, 0.01
emotion × long axis	0.19 (6124)	0.848	0.001	-0.01, 0.01
delay × emotion × long axis	-0.28 (6124)	0.778	-0.001	-0.01, 0.01
**random effects**	variance	SD	*n*	
participant (intercept)	2*e*-05	0.004	52	
stimulus set (intercept)	4*e*-06	0.002	60	
marginal R^2 / conditional R^2: 0.1 / 0.32				
**Perceptually related lures**				
**fixed effects**	*t* (df)	*p*	*β*	95% CI
intercept	2.77 (401.67)	0.006	0.005	0.001, 0.01
delay	0.03 (401.67)	0.977	7*e*-05	-0.005, 0.005
emotion	0.08 (6182)	0.938	2*e*-04	-0.004, 0.005
long axis	1.14 (6182)	0.256	0.003	-0.002, 0.01
delay × emotion	-1.19 (6182)	0.236	-0.004	-0.01, 0.002
delay × long axis	0.53 (6182)	0.599	0.002	-0.005, 0.01
emotion × long axis	0.14 (6182)	0.89	4*e*-04	-0.01, 0.01
delay × emotion × long axis	0.73 (6182)	0.467	0.003	-0.01, 0.01
**random effects**	variance	SD	*n*	
participant (intercept)	1*e*-05	0.003	52	
marginal R^2 / conditional R^2: 0.1 / 0.32				

Source data are provided as Source Data file.

Supplementary Table 15. **Analyses of delay-dependent changes in Encoding-Retrieval-Similarity in long-term memory cortices (anterior cingulate cortex, ventro-medial prefrontal cortex, inferior frontal gyrus, angular gyrus, precuneus) and sensory control regions.**

**parameters for linear mixed models**				
**long-term memory storage sites**				
**fixed effects**	*t* (df)	*p*	*β*	95% CI
intercept	8.73 (77.59)	4*e*-13	0.02	0.01, 0.02
delay	0.52 (67.12)	0.604	0.001	-0.004, 0.01
emotion	2.25 (138.12)	0.026	0.004	5*e*-04, 0.01
delay × emotion	-0.42 (3008)	0.675	-0.001	-0.005, 0.003
**random effects**	variance	SD	*n*	
participant (intercept)	6*e*-05	0.01	52	
stimulus set (intercept)	1*e*-05	0.003	60	
marginal R^2 / conditional R^2: 0.1 / 0.32				
**occipital pole**				
**fixed effects**	*t* (df)	*p*	*β*	95% CI
intercept	13.84 (70.02)	1*e*-21	0.32	0.27, 0.36
delay	0.28 (51.5)	0.784	0.01	-0.05, 0.07
emotion	0.66 (66.75)	0.513	0.01	-0.02, 0.04
delay × emotion	-1.29 (3008)	0.196	-0.01	-0.02, 0.005
**random effects**	variance	SD	*n*	
participant (intercept)	0.01	0.11	52	
stimulus set (intercept)	0.003	0.05	60	
marginal R^2 / conditional R^2: 0.1 / 0.32				
**Heschl's gyrus**				
**fixed effects**	*t* (df)	*p*	*β*	95% CI
intercept	4.20 (85.69)	0.0001	0.02	0.01, 0.020
delay	-1.40 (91.49)	0.165	-0.01	-0.02, 0.003
emotion	-0.97 (205.93)	0.333	-0.004	-0.01, 0.004
delay × emotion	1.20 (3008)	0.232	0.01	-0.004, 0.02
**random effects**	variance	SD	*n*	
participant (intercept)	2*e*-04	0.01	52	
stimulus set (intercept)	1*e*-05	0.004	60	
marginal R^2 / conditional R^2: 0.1 / 0.32				

Source data are provided as Source Data file.

Supplementary Table 16. **Analyses of delay-dependent changes in similarity between encoded images and corresponding semantically related lures in long-term memory cortices (anterior cingulate cortex, ventro-medial prefrontal cortex, inferior frontal gyrus, angular gyrus, precuneus) and sensory control regions.**

**parameters for linear mixed model**				
**long-term memory storage sites**				
**fixed effects**	*t* (df)	*p*	*β*	95% CI
intercept	9.11 (89.82)	2*e*-14	0.02	0.01, 0.02
delay	0.18 (70.43)	0.856	4*e*-04	-0.004, 0.01
emotion	0.95 (115.16)	0.345	0.002	-0.002, 0.01
delay × emotion	0.7 (3008)	0.486	0.001	-0.002, 0.01
**random effects**	variance	SD	*n*	
participant (intercept)	5*e*-05	0.01	52	
stimulus set (intercept)	2*e*-05	0.004	60	
marginal R^2 / conditional R^2: 0.1 / 0.32				
**occipital pole**				
**fixed effects**	*t* (df)	*p*	*β*	95% CI
intercept	9.11 (89.82)	2*e*-14	0.02	0.01, 0.02
delay	0.18 (70.43)	0.856	4*e*-04	-0.004, 0.01
emotion	0.95 (115.16)	0.345	0.002	-0.002, 0.01
delay × emotion	0.7 (3008)	0.486	0.001	-0.002, 0.01
**random effects**	variance	SD	*n*	
participant (intercept)	5*e*-05	0.01	52	
stimulus set (intercept)	2*e*-05	0.004	60	
marginal R^2 / conditional R^2: 0.1 / 0.32				
**Heschl's gyrus**				
**fixed effects**	*t* (df)	*p*	*β*	95% CI
intercept	3.32 (87.27)	0.001	0.01	0.01, 0.02
delay	-0.23 (93.61)	0.821	-0.001	-0.01, 0.01
emotion	0.92 (203.67)	0.357	0.004	-0.004, 0.01
delay × emotion	-1.17 (3008)	0.243	-0.01	-0.02, 0.004
**random effects**	variance	SD	*n*	
participant (intercept)	2*e*-04	0.01	52	
stimulus set (intercept)	1*e*-05	0.004	60	
marginal R^2 / conditional R^2: 0.1 / 0.32				

Source data are provided as Source Data file.

Supplementary Table 17. **Analyses of delay-dependent changes in similarity between encoded images and corresponding perceptually related lures in long-term memory cortices (anterior cingulate cortex, ventro-medial prefrontal cortex, inferior frontal gyrus, angular gyrus, precuneus) and sensory control regions.**

**parameters for linear mixed models**				
**long-term memory storage sites**				
**fixed effects**	*t* (df)	*p*	*β*	95% CI
intercept	8.7 (79.59)	4*e*-13	0.02	0.01, 0.02
delay	0.07 (67)	0.943	2*e*-04	-0.01, 0.01
emotion	1.43 (130.24)	0.155	0.003	-0.001, 0.01
delay × emotion	-0.33 (3008)	0.744	-0.001	-0.005, 0.003
**random effects**	variance	SD	*n*	
participant (intercept)	7*e*-05	0.01	52	
stimulus set (intercept)	2*e*-05	0.004	60	
marginal R^2 / conditional R^2: 0.1 / 0.32				
**occipital pole**				
**fixed effects**	*t* (df)	*p*	*β*	95% CI
intercept	13.75 (69.55)	2*e*-21	0.32	0.27, 0.36
delay	-0.06 (51.58)	0.951	-0.002	-0.06, 0.06
emotion	0.8 (67.47)	0.429	0.01	-0.02, 0.04
delay × emotion	-0.47 (3008)	0.635	-0.004	-0.02, 0.01
**random effects**	variance	SD	*n*	
participant (intercept)	0.01	0.11	52	
stimulus set (intercept)	0.002	0.05	60	
marginal R^2 / conditional R^2: 0.1 / 0.32				
**Heschl's gyrus**				
**fixed effects**	*t* (df)	*p*	*β*	95% CI
intercept	3.08 (96)	0.003	0.01	0.004, 0.02
delay	-0.22 (96)	0.828	-0.001	-0.01, 0.01
emotion	0.67 (3066)	0.503	0.003	-0.01, 0.01
delay × emotion	0.11 (3066)	0.916	0.001	-0.01, 0.01
**random effects**	variance	SD	*n*	
participant (intercept)	2*e*-04	0.01	52	
marginal R^2 / conditional R^2: 0.1 / 0.32				

Source data are provided as Source Data file.

Supplementary Table 18. **Control variables.**

**control variable**	**M (SEM)**		**two-sample *t*-test**			
	**1d**	**28d**	***t* (df)**	***p***	***d***	**95% CI**
state anxiety (STAI-S)	32.92 (0.67)	34.77 (1.43)	-1.17 (35.56)	0.250	0.33	-0.22, 0.88
trait anxiety (STAI-T)	33.19 (1.16)	32.77 (1.44)	0.23 (47.79)	0.820	-0.06	-0.61, 0.48
sleep quality (PSQI)						
global score (last 28d)	4.23 (0.39)	4.92 (0.53)	1.05 (45.59)	0.297	0.30	-0.25, 0.84
sleep quality in the last 24h	1.69 (0.12)	1.92 (0.12)	0.67 (48.15)	0.505	-0.19	-0.73, 0.36
sleep latency in the last 24h	7.44 (0.22)	7.21 (0.26)	-1.34 (49.99)	0.188	0.38	-0.17, 0.92
depressive mood (BDI II)	4.23 (0.72)	3.77 (0.74)	0.45 (49.97)	0.657	0.45	-0.67, 0.42
subjective chronic stress (TICS)	10.88 (1.06)	12.73 (1.21)	-1.15 (49.09)	0.257	0.32	-0.22, 0.87

Source data are provided as Source Data file.

Supplementary References

1. Ciric, R. *et al.* TemplateFlow: FAIR-sharing of multi-scale, multi-species brain models. *Nat. Methods* **19**, 1568-1571 (2021).
