# Supplementary material for: Time-dependent memory transformation in hippocampus and neocortex is semantic in nature
Source: Nat Commun. 2023 Sep 27;14:6037. doi: 10.1038/s41467-023-41648-1 (PMC10533832; doi:10.1038/s41467-023-41648-1)
Supplement: Supplementary file 3 — Reporting Summary [file 41467_2023_41648_MOESM3_ESM.pdf]

nature portfolio

Corresponding author(s): Lars Schwabe

Last updated by author(s): Jul 10, 2023

# Reporting Summary

Nature Portfolio wishes to improve the reproducibility of the work that we publish. This form provides structure for consistency and transparency in reporting. For further information on Nature Portfolio policies, see our Editorial Policies and the Editorial Policy Checklist.

## Statistics

For all statistical analyses, confirm that the following items are present in the figure legend, table legend, main text, or Methods section.

| n/a | Confirmed | |
|---|---|---|
| ☐ | ☒ | The exact sample size (*n*) for each experimental group/condition, given as a discrete number and unit of measurement |
| ☐ | ☒ | A statement on whether measurements were taken from distinct samples or whether the same sample was measured repeatedly |
| ☐ | ☒ | The statistical test(s) used AND whether they are one- or two-sided<br>*Only common tests should be described solely by name; describe more complex techniques in the Methods section.* |
| ☐ | ☒ | A description of all covariates tested |
| ☐ | ☒ | A description of any assumptions or corrections, such as tests of normality and adjustment for multiple comparisons |
| ☐ | ☒ | A full description of the statistical parameters including central tendency (e.g. means) or other basic estimates (e.g. regression coefficient) AND variation (e.g. standard deviation) or associated estimates of uncertainty (e.g. confidence intervals) |
| ☐ | ☒ | For null hypothesis testing, the test statistic (e.g. *F*, *t*, *r*) with confidence intervals, effect sizes, degrees of freedom and *P* value noted<br>*Give P values as exact values whenever suitable.* |
| ☒ | ☐ | For Bayesian analysis, information on the choice of priors and Markov chain Monte Carlo settings |
| ☒ | ☐ | For hierarchical and complex designs, identification of the appropriate level for tests and full reporting of outcomes |
| ☐ | ☒ | Estimates of effect sizes (e.g. Cohen's *d*, Pearson's *r*), indicating how they were calculated |

*Our web collection on statistics for biologists contains articles on many of the points above.*

## Software and code

Policy information about availability of computer code

| | |
|---|---|
| Data collection | For the pilot study, stimuli were presented using PsychpPy Version 1.90.1. Experiments for the main study were implemented using MATLAB (www.mathworks.com) with the Psychophysics Toolbox 3 extensions. MRI data were acquired using a 3T Prisma Scanner (Siemens, Germany) with a 64-channel head coil. |
| Data analysis | Behavioral analyses were performed with R version 4.0.2 (https://www.r-project.org/). The package lme4 Version 1.1.31 was used for (generalized) LMM-analyses. Post-hoc tests (t-, z- and interaction contrasts) were applied by contrasting Estimated Marginal Means of each ANOVA-model or (generalized) LMM with the Satterwaite's degrees of freedom method and Šidák correction for multiple comparisons using the R-package emmeans Version 1.7.2. Results were visualized by utilizing bar plots and individual data points with the package ggplot2 Version 3.4.2 and plotting marginal effects of generalized LMMs with the package sjPlot Version 2.8.12.<br><br>Preprocessing and first level modelling of the fMRI data was performed using SPM12 (Wellcome Trust Centre for Neuroimaging, London, UK) in MATLAB 2020b. Multivariate fMRI analyses were applied by correlating representational similarity patterns of interesting conditions on the level of participants using custom scripts in MATLAB 2020b (The Mathworks, Inc, Natick, USA). Multivariate fMRI data was further analyzed on the second level using R (see analyses of behavioral data).<br><br>Custom code to analyze the data is available at Github: https://github.com/valentinakrenz/semanticMemoryTransformation |

For manuscripts utilizing custom algorithms or software that are central to the research but not yet described in published literature, software must be made available to editors and reviewers. We strongly encourage code deposition in a community repository (e.g. GitHub). See the Nature Portfolio guidelines for submitting code & software for further information.

# Data

Policy information about availability of data

All manuscripts must include a data availability statement. This statement should provide the following information, where applicable:

- Accession codes, unique identifiers, or web links for publicly available datasets
- A description of any restrictions on data availability
- For clinical datasets or third party data, please ensure that the statement adheres to our policy

The behavioral and fMRI data generated in this study have been deposited in the Open Science Framework (OSF) database under the link: https://doi.org/10.17605/OSF.IO/W5MXR.
The raw brain imaging data are protected and are not available due to data privacy laws. The processed fMRI data are available at OSF. The Figure and Table data generated in this study are provided in the Source Data file and at OSF. Source data are provided with this paper. ROI masks used for fMRI analyses were derived from the Harvard-Oxford atlas as included in the FMRIB Software Library (https://fsl.fmrib.ox.ac.uk/fsl/fslwiki/FSL), from the WFU pick-atlas (https://www.nitrc.org/projects/wfu_pickatlas/) and from the neurosynth.org database (https://neurosynth.org/). All ROIs adapted for this study are available at the study's repository at OSF.

# Research involving human participants, their data, or biological material

Policy information about studies with human participants or human data. See also policy information about sex, gender (identity/presentation), and sexual orientation and race, ethnicity and racism.

| | |
|---|---|
| Reporting on sex and gender | We ensured a balanced representation of both males and females in our sample (N=52, 26 females and 26 males). Critically, this balance was also maintained within both of our delay groups, with each comprising 13 males and 13 females. However, our study was not designed to investigate any differences specific to either biological sex. Given that our key hypotheses revolved around the interactions between two or three variables (delay, emotion, lure type), the representation of each sex per delay group wouldn't have granted sufficient statistical power for meaningful conclusions regarding sex-specific effects. Allocation to each sex group was based on the participant's self-disclosed information. All participants provided consent for sharing of the (anonymzed) individual-level data. Each participant's sex is provided in the Source Data file and on OSF. |
| Reporting on race, ethnicity, or other socially relevant groupings | We did not assess the sociopolitical constructs of race or ethnicity nor other socially relevant groupings. |
| Population characteristics | To validate the stimulus set, we conducted a behavioral pilot study in a sample of 33 undergraduate psychology students (24 females, 9 males; age: M = 22.48 years, SEM = 0.60 years). One participant did not finish the task due to discomfort during viewing the emotionally negative stimuli, resulting in a final sample of 32 participants (23 females, 9 males; age: M = 22.53 years, SEM = 0.62 years).<br><br>Fifty-five healthy volunteers (28 males, 27 females, age: M = 24.22 years, SEM = 0.54) participated in the fMRI experiment (the main study). To avoid potential influences on memory, we included only participants without a history of any psychiatric or neurological diseases, medication intake or drug abuse in our main study. To further minimize individual laterality differences, only right-handed individuals were included in the sample. These inclusion criteria as well as any contraindications for MRI measurements were checked in a standardized interview prior to study participation. Three participants had to be excluded from the analysis because of technical failure (n = 1) or falling asleep during at least one of the MRI sessions (n = 2), resulting in a final sample of 52 participants (26 females, 26 males, age: M = 24.29 years, SEM = 0.55 years). The sample included both students and non-students, but psychology students were excluded to avoid subject-expectancy effects. To avoid potential effects, the sample was further restricted to young adults, i.e. individuals between 18 and 35 years of age.<br><br>Furthermore, we used questionnaires to assess self-reported depressive mood, anxiety, chronic stress and sleep quality and latency to control for potential influences on differences between groups. |
| Recruitment | For the behavioral pilot study, undergraduate psychology students from the University Hamburg were recruited through SONA, an online platform which provides course credit for participation in experiments. The main study employed a convenience sample, comprised of volunteers responding to online job advertisements and physical flyers. While this recruitment method might include the possibility of the sample differing from the general population in certain characteristics beyond the above stated inclusion criteria, any potential self-selection bias should be evenly distributed among our groups and, thus, should not impact the results reported in this manuscript. |
| Ethics oversight | The study protocol was approved by the ethics committee of the Medical Chamber Hamburg (PV5480) and was in accordance with the declaration of Helsinki. |

Note that full information on the approval of the study protocol must also be provided in the manuscript.

# Field-specific reporting

Please select the one below that is the best fit for your research. If you are not sure, read the appropriate sections before making your selection.

☐ Life sciences ☒ Behavioural & social sciences ☐ Ecological, evolutionary & environmental sciences

# Behavioural & social sciences study design

All studies must disclose on these points even when the disclosure is negative.

| | |
|---|---|
| Study description | Quantitative experimental study in humans using fMRI and behavioral measures. |
| Research sample | To validate the stimulus set, we conducted a behavioral pilot study in a convenience sample of 33 undergraduate psychology students (24 females, 9 males; age: M = 22.48 years, SEM = 0.60 years). One participant did not finish the task due to discomfort during viewing the emotionally negative stimuli, resulting in a final sample of 32 participants (23 females, 9 males; age: M = 22.53 years, SEM = 0.62 years).<br><br>Fifty-five healthy volunteers (28 males, 27 females, age: M = 24.22 years, SEM = 0.54) participated in the fMRI experiment (the main study). To avoid potential influences on memory, we included only participants without a history of any psychiatric or neurological diseases, medication intake or drug abuse in our main study. To further minimize individual functional laterality differences, only right-handed individuals were included in the sample. These inclusion criteria as well as any contraindications for MRI measurements were checked in a standardized interview prior to study participation. Three participants had to be excluded from the analysis because of technical failure (n = 1) or falling asleep during at least one of the MRI sessions (n = 2), resulting in a final sample of 52 participants (26 females, 26 males, age: M = 24.29 years, SEM = 0.55 years). This concenience sample included both students and non-students, but psychology students were excluded to avoid subject-expectancy effects. To avoid potential age-related differences in memory performance between groups, we further included only young adults, i.e. individuals between 18 and 35 years of age, into our sample. Furthermore, we used questionnaires to assess self-reported depressiveness, anxiety, chronic stress and sleep quality and latency to control for potential influences on differences between groups. |
| Sampling strategy | We applied a convenience sampling procedure with a pseudo-randomized allocation to delay-groups. The final sample size is in line with previous fMRI studies on time-dependent memory-transformation processes and a sensitivity analysis using MorePower Version 6.0.45 confirmed that this sample size is sufficient to detect a medium-sized effect for our primary behavioral effect of interest reflected in a 2 (delay) × 3 (lure type) × 2 (emotion) mixed ANOVA with a power of 0.80 ($\alpha$ = 0.05). |
| Data collection | The behavioral pilot study was computer-based using PsychoPy 2 Version 1.90.1. During the free recall task, participants named all remembered items from the encoding task in as much detail as possible while the experimenter ticked off the remembered items from a list and an audio recording was made for further reference. All other behavioral tasks were computer-based using MATLAB with the Psychophysics Toolbox 3 extensions. Demographic and questionnaire data were gathered using electronic tablets.<br><br>Throughout all behavioral tasks, only the experimenter and the participant were present. Encoding and recognition testing was conducted within the confines of the MRI scanner. Due to the logistical requirements of the study, wherin the experimenter usually coordinated the scheduling of the three experimental days, keeping the experimenter unaware of each participant's delay group was not feasible. Additionally, as most of the experimenters were involved in this research project as part of their undergraduate studies, a full blinding to the research hypothesis was not possible. To reduce potential influences from the experimenter, participants were predominatly given instructions in written form. Additionally, the experimenters received comprehensive training in the starndardized administration of the experiment. They were also equipped with in-depth written guidlines, which included detailed, pre-formulated verbal instructions to be used when necessary, further ensuring the standardization and consistency of the experiment. |
| Timing | Data collection took place between January 2019 and February 2020. |
| Data exclusions | Three participants had to be excluded from the analysis because of technical failure (n = 1) or falling asleep during at least one of the MRI sessions (n = 2). |
| Non-participation | One participant of the behavioral pilot study did not finish the task due to discomfort during viewing the emotionally negative stimuli. |
| Randomization | Depending on the participant's availability, they completed experimental Day 2 either 1 day or 28 days after the first experimental day. Therefore, assignment to the delay condition was pseudo-randomized. |

# Reporting for specific materials, systems and methods

We require information from authors about some types of materials, experimental systems and methods used in many studies. Here, indicate whether each material, system or method listed is relevant to your study. If you are not sure if a list item applies to your research, read the appropriate section before selecting a response.

## Materials & experimental systems

| n/a | Involved in the study |
|---|---|
| ☒ | ☐ Antibodies |
| ☒ | ☐ Eukaryotic cell lines |
| ☒ | ☐ Palaeontology and archaeology |
| ☒ | ☐ Animals and other organisms |
| ☒ | ☐ Clinical data |
| ☒ | ☐ Dual use research of concern |
| ☒ | ☐ Plants |

## Methods

| n/a | Involved in the study |
|---|---|
| ☒ | ☐ ChIP-seq |
| ☒ | ☐ Flow cytometry |
| ☐ | ☒ MRI-based neuroimaging |

# Magnetic resonance imaging

## Experimental design

Design type

Task-based fMRI with an event-related design.

Design specifications

The encoding task consisted of three consecutive runs of 7min each in which the same 60 stimuli were randomly presented, i.e. each stimulus was presented once in each run. In each trial, a picture was presented for 3s followed by a jittered fixation period for 4±1s.
The recognition task was separated into three consecutive runs with 80 trials each. During the recognition test, participants saw the 60 pictures that were presented on Day 1 ('old') and 60 pictures that were new but semantically related to the old pictures, 60 pictures that were perceptually related to the old pictures and 60 pictures that were neither perceptually nor semantically related to the old pictures. In each trial, a picture was presented for 3s followed by a rating scale, which was presented for max. 3s or until a response was given. Between trials, a jittered fixation cross was presented for 4s±1s.
During the semantic relatedness rating task, participants were presented with each originally encoded ('old') picture next to each corresponding (perceptually related, semantically related, unrelated) lure and rated the perceptual and semantic relatedness of each picture pair on a scale reaching from 1 ('not related) to 10 ('very related'). During the behavioral pilot and the main study, participants rated the perceptual and semantic relatedness of 210 or 180 picture pairs, respectively.

Behavioral performance measures

Missing responses during encoding and recognition were examined to control for the alertness during encoding in the MRI scanner. To control for potential group differences right after encoding, immediate memory performance was assessed in a free recall task. As an indicator of memory performance during recognition testing, we compared the hit-rate (i.e. the correct indorsement of old items as 'old') between groups and conditions. As an indicator of memory specificity, our main research question, false alarms (i.e. the incorrect endorsement of new items as 'old') between the three lure types were compared.

## Acquisition

Imaging type(s)

Functional MRI and structural MRI for coregistration of functional images.

Field strength

3T.

Sequence & imaging parameters

A magnetic (B0) field map was assessed to unwarp the functional images (TR=634ms, TE1=4.92ms, TE2=7.38ms, 40 slices, voxel size=2.9x2.9x3.0mm3, FOV=224mm). For the functional scans, T2*-weighted echo planar imaging sequences were used to obtain 2mm thick transversal slices (TR=2000ms, TE=30ms, flip angle=60°, FOV=224). Additionally, a high-resolution T1 weighted anatomical image (TR=2500 ms, TE=2.12 ms, 256 slices, voxel size =0.8x0.8x0.9mm3) was collected for coregistration of the functional scans.

Area of acquisition

Whole-brain scan.

Diffusion MRI ☐ Used ☒ Not used

## Preprocessing

Preprocessing software

Preprocessing was performed with SPM12. The images were first realigned and unwarped using the field maps, then coregistered to the structural image followed by a normalization to Montreal Neurological Institute (MNI) space. The multivariate fMRI analyses were applied on unsmoothed data.

Normalization

Images were spatially normalized using SPM12's unified segmentation.

Normalization template

Data were normalized into standard stereotactic (MNI) space using SPM12's standard template (IXI549Space).

Noise and artifact removal

SPM12 realign and unwarp was used to correct for motion artifacts and geometric distortions.

Volume censoring

None.

## Statistical modeling & inference

**Model type and settings**

On the first level, the functional MRI data was analyzed using general linear modeling (GLM) as implemented in SPM12. For our multivariate analyses, each individual trial of the encoding and recognition task was modelled as an individual regressor convolved with a hemodynamic response function along with six session-constants in one GLM per subject using SPM12. A high-pass filter of 128s was used to remove low-frequency drifts and serial correlations in the time series were accounted for using an autoregressive AR(1)-model.

**Effect(s) tested**

Hippocampal memory reinstatement analyses: Time-dependent changes in similarity (Fisher z-transformed r-values) between activation patterns at encoding and memory test (Encoding-Retrieval-Similarity, ERS) were analyzed by means of trial-wise linear mixed models (LMMs) with the factors delay (1d vs. 28d), emotion (neutral vs. negative), long axis (anterior vs. posterior) and their interactions as fixed effects and the random intercept of participants and stimuli. Further, we followed up whether the observed delay-dependent increase in left posterior hippocampal ERS was associated with a decrease in specificity of the reinstated memories. To this end, we analyzed the occurrence of a FA for a semantically related or perceptually related lure by means of binomial generalized LMMs with emotion (neutral vs. negative), delay (1d vs. 28d), ERS and their interaction as fixed effects and the random intercept of participants and stimuli.
Model-Based Analysis: Z-transformed rho-values indicating the fit of neural representational patterns to conceptual models were subjected to mixed ANOVAs with the factors delay (1d vs. 28d), emotion (neutral vs. negative) and conceptual model (1: 'old items are distinct' vs. 2: 'old and semantically related items are similar and old' vs. 3: 'perceptually related items are similar').

**Specify type of analysis:** ☐ Whole brain ☒ ROI-based ☐ Both

**Anatomical location(s)**

Anatomical masks for the aCC, precuneus, angular gyrus (left and right), the occipital pole and Heschl's gyrus (left and right) were derived from the Harvard-Oxford atlas using a probability threshold of 50%. For the inferior frontal gyrus (IFG) and ventromedial prefrontal cortex (vmPFC), a sphere with 20mm radius was used that was centered on the peak voxel (x = -50, y = 16, z = 12) derived from 386 imaging studies reporting 'IFG' and on the peak voxel (x = -2, y = 46, z = -8) derived from 199 imaging studies reporting 'vmPFC', respectively, as determined by meta-analyses conducted on the neurosynth.org platform (status 02/06/2022). Anatomical masks of the anterior and posterior hippocampus (left and right) were derived using the WFU pick-atlas.

**Statistic type for inference**

(See Eklund et al. 2016)

Does not apply (no univariate analyses).

**Correction**

To minimize the effects of multiple testing, all relevant regions of interest were included in the statistical models for hypothesis testing. If testing was repeated for both heminspheres, Bonferroni Correction was applied.

## Models & analysis

| n/a | Involved in the study |
|---|---|
| ☒ | ☐ Functional and/or effective connectivity |
| ☒ | ☐ Graph analysis |
| ☐ | ☒ Multivariate modeling or predictive analysis |

**Multivariate modeling and predictive analysis**

For our model-based RSA-analyses, each trial's activation pattern across voxels was correlated (Pearson's r) with the activation patterns of each other trial during memory testing, separately for each emotionality category. Next, we computed the mean pattern similarity for comparisons within each of the three runs and for each between-run combination (run 1 and run 2, run 2 and run 3 or run 3 and run 1). Those run-related pattern similarities where then subtracted from each correlation estimate of the corresponding run-combination to account for inflated correlations as a function of temporal proximity between scans. In the resulting 120 × 120 RSMs, each combination of trials was placed in the respective cells, ordered by stimulus type (Figure 4A, left panel). The resulting neural RSMs were compared to three theoretical model RSMs, each predicting different similarity patterns between the four stimulus categories at recognition testing: similar representations for old pictures that are distinct from patterns for all novel stimuli (model 1: 'old items are distinct from all lures'), similar representations between old items and semantically related lures which are distinct from perceptually related and unrelated lures (model 2: 'old and semantically related items are similar') and a model that expects similar representations between old items and perceptually related lures which are distinct from semantically related and unrelated lures (model 3: 'old and perceptually related items are similar'). We computed Spearman's rank correlation coefficient for each single-subject RSM and the conceptual models as we did not assume a direct linear match between the compared RSMs. The resulting rho-values were further z-transformed before subjecting them to statistical analyses in R.

For our memory reinstatement analysis, activation patterns across all encoding runs, as a reliable indicator of encoding-related activity, were correlated (Pearson's r) with activation patterns while viewing the same (old) items at memory testing. Resulting correlation-values were Fisher z-transformed before statistical analyses in R.
